# Calcification process dynamics in coral primary polyps as observed using a calcein incubation method

**DOI:** 10.1016/j.bbrep.2017.01.006

**Published:** 2017-01-24

**Authors:** Yoshikazu Ohno, Akira Iguchi, Chuya Shinzato, Mikako Gushi, Mayuri Inoue, Atsushi Suzuki, Kazuhiko Sakai, Takashi Nakamura

**Affiliations:** a Marine and Environmental Sciences Course, Graduate School of Engineering and Science, University of the Ryukyus, Senbaru 1, Nishihara, Okinawa 903-0213, Japan; b Department of Bioresources Engineering, National Institute of Technology, Okinawa College, 905 Henoko, Nago, Okinawa 905-2192, Japan; c Marine Genomics Unit, Okinawa Institute of Science and Technology Graduate University, Onna, Okinawa 904-0495, Japan; d Graduate School of Natural Science and Technology, Okayama University, 3-1-1 Tsushima-naka, Okayama 700-8530, Japan; e Geological Survey of Japan, National Institute of Advanced Industrial Science and Technology (AIST), 1-1-1 Higashi, Tsukuba, Ibaraki 305-8567, Japan; f Sesoko Station, Tropical Biosphere Research Center, University of the Ryukyus, 3422 Sesoko, Motobu, Okinawa 905-0227, Japan; g Japan Science and Technology Agency (JST)/Japan International Cooperation Agency (JICA) SATREPS, Tokyo, Japan

**Keywords:** Primary aposymbiotic coral polyp, Sub calicoblastic medium, Calcification, Live imaging, Calcein

## Abstract

Calcification processes are largely unknown in scleractinian corals. In this study, live confocal imaging was used to elucidate the spatiotemporal dynamics of the calcification process in aposymbiotic primary polyps of the coral species *Acropora digitifera*. The fluorophore calcein was used as a calcium deposition marker and a visible indicator of extracellular fluid distribution at the tissue-skeleton interface (subcalicoblastic medium, SCM) in primary polyp tissues. Under continuous incubation in calcein-containing seawater, initial crystallization and skeletal growth were visualized among the calicoblastic cells in live primary polyp tissues. Additionally, the distribution of calcein-stained SCM and contraction movements of the pockets of SCM were captured at intervals of a few minutes. Our experimental system provided several new insights into coral calcification, particularly as a first step in monitoring the relationship between cellular dynamics and calcification *in vivo*. Our study suggests that coral calcification initiates at intercellular spaces, a finding that may contribute to the general understanding of coral calcification processes.

## Highlights

• Coral calcification dynamics were observed by using aposymbiotic primary polyps.
• CaCO_3_ deposits during skeletal formation were visualized by calcein fluorescence labeling and time-lapse confocal microscopy.
• Periodic pulsing activity of the pockets of subcalicoblastic medium (SCM) was detected.

## 1 Introduction

Coral skeletons contribute to maintain a high level of biodiversity in ecosystems associated with coral reef formations. Although numerous studies have investigated the physiological and molecular aspects of coral calcification mechanisms, the actual calcification mechanism, particularly the initial nucleation and subsequent calcium carbonate (CaCO_3_) crystal deposition, has been debated for more than a century [1]. Mechanistic aspects of this process, particularly those concerning calcium transport at the level of the calcifying cells (i.e., calicoblastic cells), remain unclear.

One of the major approaches for visualizing calcification is the microscopic observation of calcifying sites. For example, electron microscopic technique-based studies clarified the fine morphological features of the calcifying interface and described various nano-crystals, granular skeletal structures, and other components [2], [3]. Isotope analyses, which include calcifying fluid pH measurement using a boron isotope [4] and calcium transmembrane transport evaluation using a calcium isotope [5], [6], have also been used to investigate calcification mechanisms in corals. These techniques have provided additional details about coral calcification, but are restricted to static conditions for coral calcification.

Fluorescence live imaging technique with X-ray microanalysis was applied to the study of calcium transport and the storage from the seawater around the calcifying sites in coral tissues at early life stages (planula larvae and settled primary polyps) [7]. Recently, confocal live imaging has recently been used to investigate calcification mechanisms in live coral tissue; this technique enables us to observe dynamic coral calcification processes. For example, live imaging using fluorescent dyes has been used to visualize the pH of the subcalicoblastic medium (SCM) and clarify the biological responses of live coral tissues to ocean acidification [8], [9], [10]. These techniques have provided new insights into several important aspects of calcification, such as pH elevation in SCM and responses of the pH_SCM_ to seawater acidification.

Calcein (fluorescein-3,3′-bismethylimino-discetic acid), which is a fluorescent calcium indicator that binds to calcium ion, is incorporated into precipitating calcium carbonate crystals. Calcein is a water-soluble molecule that cannot penetrate the cell membrane. These characteristics have led to the use of calcein for hard tissue staining in a wide variety of marine organisms (e.g., fish otolith [11]; foraminiferan shells [12]; coral skeletons [13]). In particular, the calcein has been used to image coral crystal growth [8], [9], [13], [14] and SCM areas in corals on glass substrates [8]. Furthermore it has been used to image the intercellular spaces in corals [13].

Previous studies have demonstrated that short-term incubation with calcein did not appear to affect coral growth [15]; accordingly, calcein has been recommended instead of alizarin and Sr for tracing skeletal growth in some shellfish species [16], [17]. The effect of calcein on the incorporation of Sr and Mg into calcite has been investigated in foraminifera, and these studies found that calcein did not affect the incorporation of these elements [18]. Additionally, calcein has few adverse effects on benthic foraminifera even during long-term exposure (4–5 weeks)[19]. Thereby, we hypothesize that continuous incubation of coral cultures in calcein is possible, and fluorescent imaging of corals subjected to long-term calcein exposure will enable a real-time visual observation of coral calcification, particularly the initiation of skeletal growth.

In the present study, we used a confocal imaging system to directly observe coral calcification processes *in vivo*. Based on the above-described merits, calcein was selected as a fluorescent marker for continuous skeletal growth monitoring. We also used an experimental system using coral primary polyps of *Acropora* species obtained at mass coral spawning events, which allowed us to observe initial calcification after the settlement of coral planulae [20]. *Acropora* planulae initiate calcification shortly after settlement by forming calcium carbonate (CaCO_3_) structures at the interface between the larval tissues and substrate [21]. The simple morphology and the lack of symbiotic algae of these polyps render the calcification process easily visible. The present study proposes a detailed observational method of coral calcification in live tissues of early life stages which enables us to deeply understand the physiological aspects of coral calcification.

## 2 Materials and methods

### 2.1 Sample preparation

The scleractinian coral *Acropora digitifera*, which is one of the most common species in the Ryukyu Islands of Japan [22], was used in this study. Gravid colonies of *A. digitifera* were collected from a fringing reef at Sesoko Island, Motobu-cho in Okinawa, Japan. In addition, several colonies of a cryptic *A. digitifera* species (*Acropora* sp.1) [23] were also collected at Bise, Motobu-cho in Okinawa, Japan. The colonies were kept in a running seawater tank under natural light conditions at Sesoko Station, Tropical Biosphere Research Center, University of the Ryukyus, Okinawa, Japan. Coral spawning occurred at night around the time of the full moon in the spring and summer seasons of 2013–2015. Gametes were collected after spawning as described by Morita et al. (2006) [24]. Primary polyps were prepared by inducing settlement of the planula larvae (3–30 days old) using the coral metamorphosis inducer peptide Hym-248 [25]. Hym-248 induces the synchronous metamorphosis and settlement of *Acropora* planulae, and is a useful tool for studies of *Acropora* larval metamorphosis [26]. Approximately 5–10 larvae were placed in a glass-based dish (No. 1S, thickness: 0.15–0.18 mm; IWAKI Glass, Tokyo, Japan) with 40 µL droplet of filtered seawater (FSW: pore size 0.22 µm). About 4–6 droplets were made on the surface of the glass-based dish. Next, a 10-µL aliquot of 2×10−4 M Hym-248 in FSW was added in each droplets and the larvae were incubated for 2 h to induce metamorphosis. Finally, approximately 10–20 larvae were settled on a glass-based. Larvae that settled on the seawater surface and the side of the glass-based dish were removed.

### 2.2 Calcein

Calcein was purchased from Sigma-Aldrich (St. Louis, MO, USA). A stock solution containing 2 gL^−1^ calcein was prepared in distilled water and buffered to pH 6 using sodium bicarbonate to enhance the solubility of calcein [13]. This solution was then diluted in FSW buffered to pH 8.1 (total pH scale) with NaOH to obtain a final concentration of 100 µM (FSW-calcein: salinity of approximately 35). After 2-h incubation with Hym-248, the solution was made up to 2000 µL with FSW-calcein. The pH was measured using a portable pH meter (D-71; Horiba, Ltd., Kyoto, Japan) as a total scale with a precision of±0.01 pH units. The detail effects of long-term calcein incubation on coral polyp were shown in Supplementary Fig. 1 and Supplementary Fig. 2.

### 2.3 Confocal microscopy

In this experiment, we mainly used a spinning-disk confocal imaging system equipped with an Eclipse Ti-U inverted epifluorescence microscope (Nikon, Tokyo, Japan), hand-made reflection light, CSU-X1 laser-scanning unit (Yokogawa, Tokyo, Japan), and ImagEM C9100-13 electron-multiplying charge-couple device (EM-CCD) camera (Hamamatsu Photonics, Hamamatsu, Japan). The system was operated by a Hamamatsu Photonics AQUACOSMOS/RATIO system. During time-lapse confocal imaging, the exposure time was set to 200 msec, and calcein signals were recorded at 10-min intervals using a 488 nm excitation light and a 505–540 nm bandpass filter. We used another confocal system (A+confocal microscope system; Nikon) that was equipped with a high-resolution galvano scanner and operated by NIS Elements software (Nikon) to visualize crystallization at the cellular level. Calcein was excited at 480 nm and fluorescence was detected at 510–530 nm. Each individual specimen was placed on a glass-based dish and filled with 2 mL of FSW-calcein (100 µM) at room temperature (approximately 26 °C). To set the Z=0 µm on the surface of the glass substrate, we marked the crystals on the glass cover slip [8].

## 3 Results and discussion

### 3.1 Calcein staining patterns in coral primary polyps

**Fig. 1:**
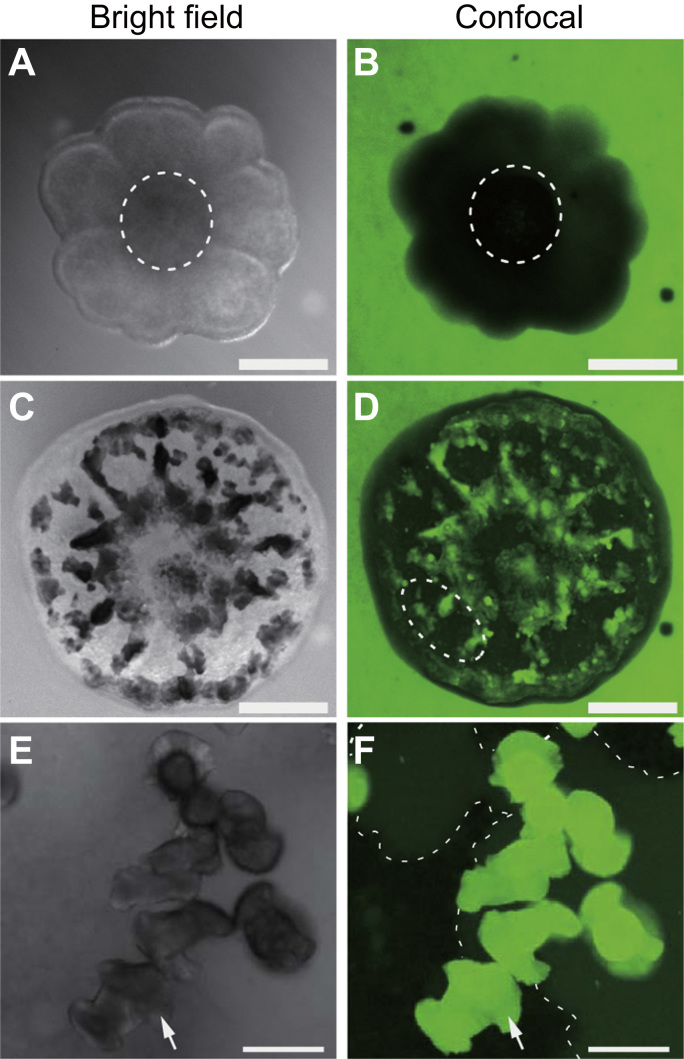
Confirmation of coral skeletons and tissue staining patterns with or without calcein. Specimens were incubated in calcein-containing seawater during the experiment. **(A)** Bright-field image of a primary polyp 12 h after incubation. Scale bar: 200 µm. **(B)** Confocal image of the same position in **(A). (C)** Bright-field image of the primary polyp in **(A)** at 56 h after incubation. The black area indicates the coral skeleton. Scale bar: 200 µm. **(D)** Confocal image of the same position in **(C)**. Dotted lines indicate the area approximately quarter to half from the periphery of the primary polyp. The coral skeleton was stained using calcein (green). **(E)** High-magnification bright-field image of coral skeletons from the polyps in **(A–D)** at 24 h after incubation. The white arrow indicates a dumbbell-shaped crystal. Scale bar: 20 µm. **(F)** Confocal image of the same position in **(E)**. White arrow indicates a crystal on the surface of the glass-based dish. Dotted lines indicate the periphery of the subcalicoblastic medium (SCM). The black area indicates the bottom of the coral tissue.

**Fig. 2:**
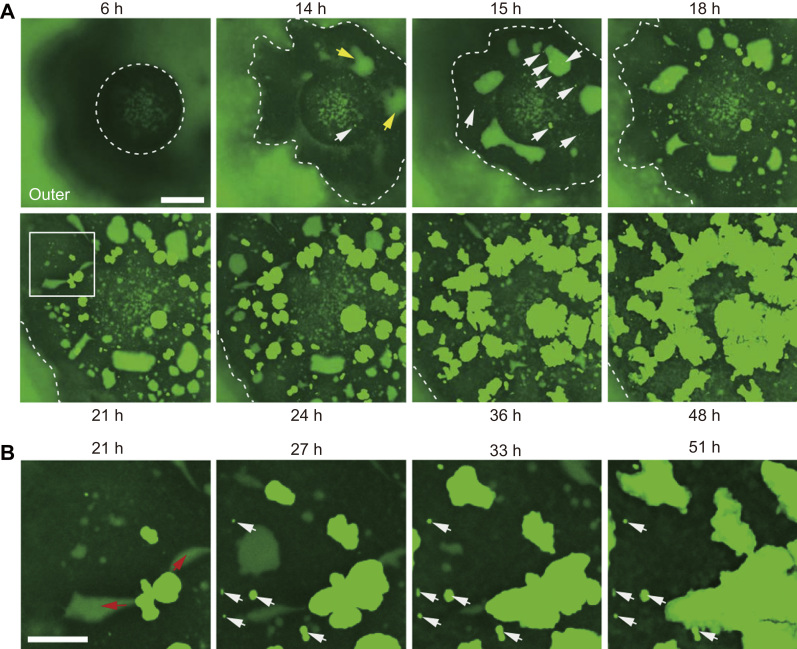
An observation in calcein-containing filtered seawater (FSW-calcein) at 6 h after the addition of Hym-248. The numbers in the upper and bottom parts of the panels indicate the recording times. Dotted lines indicate the periphery of the attached bottom part of the coral primary polyp. **(A)** Time series of images showing the developmental process of the primary polyp at the bottom (coral skeletal growth: bright green); distribution of calcein (green); and coral tissues (black). White arrows indicate the initial crystallization (12–15 h). SCMs are indicated by yellow arrows. Scale bar: 100 µm. **(B)** High-magnification images of the square area denoted by a white line in **(A)**. Red arrows indicate the direction of skeletal growth along the narrow SCM pocket. White arrows indicate crystals without growth. Scale bar: 50 µm.

**Fig. 3:**
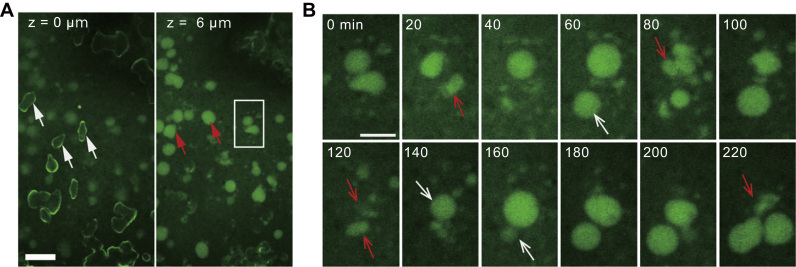
An observation initiated 5 h after the addition of calcein (24 h after the addition of Hym-248). **(A)** Confirmation of crystal (white arrows in the left image, z=1 µm from the bottom) and SCM distribution (red arrows in the right image, z=6 µm from the bottom). Scale bar: 20 µm. **(B)** Time-lapse image showing a series of SCM pockets contractile movements in the area denoted by a white line in **(A)**. The numbers in the upper left of each panel indicate the recording times. Red and white arrows indicate contraction and expansion of SCM pockets, respectively. Scale bar: 10 µm.

**Fig. 4:**
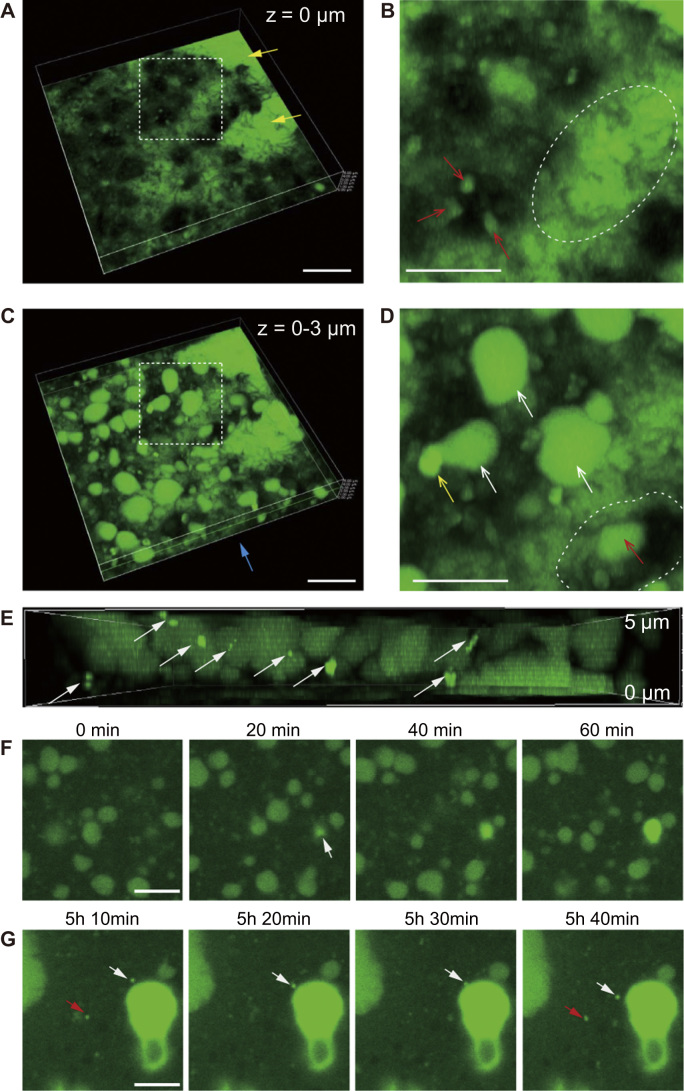
Vertical distribution of SCM, floating crystals, and calicoblastic cells. **(A)** High-magnification 3-dimensional image of the bottom of the coral primary polyp 24 h after the addition of Hym-248. The vertical height of this image is 5 µm. This image highlights the bottom of the tissue. Yellow arrows indicate relatively larger SCM pockets. The black area represents calicoblastic cells. Scale bar: 10 µm. **(B)** High-magnification image of the area enclosed by the white dotted line in **(A)**. Red arrows indicate the FSW-calcein distribution inside the cell (black). The area encircled by the white dotted line indicates dark thread-like structures among the cells. Scale bar: 5 µm. **(C)** Three-dimensional image at 0–3 µm from the bottom of the polyp, at the same position as in **(A)**. Blue arrow indicates the directions of observation of the cross-section image in **(E)**. Scale bar: 10 µm. **(D)** High-magnification image of the area enclosed by the white dotted line in **(C)**. White arrows indicate smaller SCM pockets. The yellow arrow indicates a putative nascent crystal. The red arrow indicates SCM pockets that appears to be surrounded by a calicoblastic cell (enclosed by dotted white line). Scale bar: 5 µm. **(E)** Image cross-section of **(C)**. White arrows indicate putative nascent crystals. SCM distribution is visible as darker green areas. **(F)** Time series of high magnification images reveal the initial skeletal growth, with a focus on the outer to intermediate areas in a primary polyp. Observation began 18 h after Hym-248 addition. The white arrow indicates an emerging crystal. Scale bar: 5 µm. **(G)** White and red arrows indicate floating putative nascent crystals at the bottom of the coral tissue in the same position as in **(F)**. Scale bar: 5 µm.

To investigate whether the coral skeleton could be stained by calcein during continuous incubation, we examined the calcein staining patterns in primary polyps according to skeleton formation. Bright-field images that were acquired at 6 h after Hym-248 addition showed that the bottoms of the primary polyp tissues had yet not formed skeletons (Fig. 1A). Confocal images using 488 nm (blue light) excitation revealed the distribution of fluorescent calcein-FSW only around the coral primary polyp, suggesting that calcification had not yet been initiated at this stage (Fig. 1B). Although corals have been reported to exhibit cellular autofluorescence [13], [27], the autofluorescence of the primary polyp was much weaker than the bright green fluorescence emitted by dissolved calcein in seawater (Supplementary Fig. 4). Therefore, we considered the effect of autofluorescence to be minimal in our calcein-based observation. At 56 h after the addition of Hym-248, a skeleton was produced at the bottom of the primary polyp tissue (Fig. 1C). Primary polyps were continuously incubated in calcein-FSW, thus allowing visualization of the coral skeleton as a bright green structure under blue light excitation because of the continuous accumulation of calcein during skeletal formation (Fig. 1D). The region approximately quarter to half from the periphery (Fig. 1D; white dotted line) indicated the observation area in Fig. 2, Fig. 3, Fig. 4 in different individuals of Fig. 1D. High-magnification bright-field images revealed dumbbell-shaped crystals (Fig. 1E; white arrow) similar to the structures described previously in a primary polyp of *Pocillopora damicornis* [28].

Live confocal imaging highlighted the calcein-stained CaCO_3_ crystals (Fig. 1F; white arrow) and aragonite crystalline fibers (Supplementary Fig. 3). In high magnification images, the center of calcification (Supplementary Fig. 3A; red arrows), cracks, and edges of crystals with aragonite fibers were clearly visible (Supplementary Fig. 3B, white arrows). Using our experimental method, we could confirm the existence of calcein-FSW containing SCM (namely, SCM pocket, Fig. 1F; dotted lines) and distinguish coral tissue, which appeared as black areas. However SCM pockets could not be observed using conventional bright-field microscopy (Fig. 1E). Similar observations were described in a report of a calcein-labeling method for adult organisms of the coral *Stylophora pistillata* [9], [13]. Therefore, the crystal formation and occurrence of SCM pockets around calcification sites observed in our study may be common features among coral taxa.

### 3.2 Observations of crystal development and SCM dynamics

To further understand the detailed calcification process in the context of long-term calcein-FSW incubation, we attempted to visualize morphological changes in primary polyps with a simultaneous focus on the relationship between crystal development and SCM pockets dynamics (Fig. 2A; also see supplementary movie S1). At 6 h after metamorphosis, the bottoms of the primary polyps had attached to the glass substrate (Fig. 2A, dotted circle area). At that time point, no bright calcein crystals or SCM pockets were visible within the primary polyp tissues (n=5). After a 14-h incubation, bright areas indicating crystal formation (Fig. 2A, white arrows) and large SCM pockets (approximately 50–60 µm in diameter; Fig. 2A, yellow arrows) emerged in accordance with the expansion of the attached area and crystals were gradually developed. Between 21 and 48 h of incubation, the areas of SCM were replaced with crystals. High magnification images revealed that the tips of these crystals extended toward the SCM pockets (Fig. 2B, red arrows).

Supplementary material related to this article can be found online at doi:10.1016/j.bbrep.2017.01.006.

The following is the Supplementary material related to this article Movie S1.

Movie S1 Time-lapse image of Fig. 2..

The functions of the SCM pockets are still unclear, however, we presume that the skeletal growth in corals may be coordinated by SCM distribution. The pH of SCM is reported to exceed that of seawater, and this is expected to correlate with an increased concentration of carbonate ion (CO_3_^2−^) and promote the precipitation of CaCO_3_ [8], [9]. Thus, skeletal development would be physiologically controlled by SCM distribution. It is worth noting that crystal formation was variable: some crystals grew continuously whereas others ceased to grow at smaller sizes (Fig. 2B, white arrows). We cannot address the differences in crystal growth at this point. Analyzing the content of the organic matrix during calcification may explain the heterogeneity of crystal development.

The dumbbell-shaped crystals, which were distributed throughout the outer to intermediate areas in individual polyps (e.g., Fig. 1D; white dotted line), were located only on the surface of the glass substrate (Fig. 3A, white arrows), whereas small areas of SCM (approximately 10 µm in diameter) were distal to the substrate (Fig. 3A, red arrows). In these small SCM pockets, contraction movements were also observed at intervals of a few seconds (Fig. 3B; red and white arrows indicate contracting and expanding areas of SCM pockets, respectively; also see supplementary movie S2) at a micrometer scale above the glass substrate. Two compartmentalized small SCM pockets had adhered to their boundaries (0 min). After 20 min, one of these SCMs had contracted (Fig. 3B; red arrow); this area recovered after 60 min (Fig. 3B; white arrow). These contraction movements were observed in all small SCM pockets. However, large SCM pockets (e.g., Fig. 2A; red arrows) did not disappear, even after several hours. These contraction movements are thought to provide movement of fluid in the SCM pockets, although further analyses are needed to clarify the roles of these movements.

Supplementary material related to this article can be found online at doi:10.1016/j.bbrep.2017.01.006.

The following is the Supplementary material related to this article Movie S2.

Movie S2 Time-lapse image taken 6 µm above the substrate in Fig. 3A..

### 3.3 Vertical observations of SCM pockets and putative nascent crystals

The calcein incubation method showed not only the distribution of the SCM but also the floating green particles in coral calcifying tissue. Because fluorophore calcein accumulation shows the evidence of existing calcium carbonate crystals, we assumed that these green particles are putative nascent crystals. To better understand the 3-dimensional calcification process, we vertically observed the putative nascent crystals and related SCM pockets movement at 18 h after inducing settlement. Relatively large areas of SCM were located at the glass substrate (Fig. 4A, yellow arrows). High-magnification images (Fig. 4A, dotted square area) revealed the form of coral calicoblastic cells (in black) with thread-like structures on their surfaces (Fig. 4B, dotted circle area). Seemingly, calcein fluorescent signal detected not only SCMs but also inside of the calicoblastic cells (Fig. 4B and D: red arrows; also see supplementary movie S3). Although, we could not obtain clear image of calicoblastic cells (i.e. membrane staining) at this stage, the image is reminiscent of the fluid endocytosis (pinocytosis) pathway in these calicoblastic cells. It is known that lateral calicoblastic cell membranes are highly interdigitated, with cells often appearing to overly each other [2]. However, the positional relationship among calcifying medium, crystals, and calicoblasitic cells is still obscure. Obtaining the detailed view of the area of calicobrastic cell membrane is essential for understanding the initial calcification process (i.e. crystal nucleation and growth).

Supplementary material related to this article can be found online at doi:10.1016/j.bbrep.2017.01.006.

The following is the Supplementary material related to this article Movie S3.

Movie S3 Stacking reconstruction image of Fig. 4A and C..

We also observed the relatively small-volume SCM pockets (Fig. 4E; white arrows) and putative crystals measuring several micrometers in diameter (Fig. 4D; yellow arrow) emerged 0–3 µm above the glass substrate. A cross-section of coral primary polyps revealed that these small SCM pockets and putative crystals were not attached to the glass substrate (Fig. 4E). Continuous observation also implied that the initially formed crystals had developed among calicoblastic cells at several sites within the tissues (Fig. 4F; 5 µm above the glass substrate, white arrows), suggesting that the initial crystallization occurred in some spaces within the coral tissue. Bright floating particles measuring <1 µm in diameter (i.e., putative nascent crystals) were observed at 5 h after the observation began (Fig. 4G; white arrows). Additionally, new particles emerged and disappeared in the same imaging areas (Fig. 4G, red arrows). It seems that these small putative crystals could travelling in the coral tissue. Motionless putative nascent crystals were observed in individual polyps at the glass substrate interface (Supplementary Fig. 4B, 4C). These smaller crystals did not grow in the intercellular space, although dumbbell-shaped crystals continued to grow in these imaging areas. These putative smaller crystals were morphologically similar to the rod-shaped crystals that were considered to indicate calcitic mineralogy [29].

More detailed analysis would be necessary to determine the fine structure, crystal type, and developmental process of nascent crystals in coral calcifying tissue. When we used fixed samples for X-ray microanalysis, unexpected crystallization may be observed. Thus, we cannot determine if the putative nascent crystals (from several hundred nanometers to several micrometers in diameter) in coral tissue were aragonite or calcite at this stage. A recent study demonstrated initial CaCO_3_ nucleation by *in situ* TEM measurement technique [30] and found that multiple nucleation pathways simultaneously exist. However, it is almost impossible to apply *in situ* TEM to coral calcification in a noninvasive manner. Our calcein incubation method has the potential to visualize initial crystallization on a submicron scale, but utilizing other methods such as in situ TEM measurement would be necessary to analyze CaCO_3_ crystal types in detail.

In summary, the present study successfully investigated the spatiotemporal dynamics of calcification in aposymbiotic coral primary polyps. This study was the first to successfully observe the process of crystal development, contractile movements of SCM pockets during coral calcification by long-term calcein incubation method. Further studies involving the application of our live imaging method should facilitate the visualization of calcification processes in corals, including the precipitation of organic matrix during calcification, using staining reagents (e.g., acridine orange, fluorescent antibodies). These studies will contribute further to our general understanding of biocalcification in corals.

## Footnotes

Appendix A Transparency document associated with this article can be found in the online version at http://dx.doi.org/10.1016/j.bbrep.2017.01.006.

## References

[1] Allemand D., Ferrier-Pagès C., Furla P., Houlbrèque F., Puverel S., Reynaud S. (2004). Biomineralisation in reef-building corals: from molecular mechanisms to environmental control. Comptes Rendus Palevol.

[2] Clode P.L., Marshall A.T. (2002). Low temperature FESEM of the calcifying interface of a scleractinian coral. Tissue Cell.

[3] Clode P.L., Marshall A.T. (2003). Skeletal microstructure of *Galaxea fascicularis* exsert septa: a high-resolution SEM study. Biol. Bull..

[4] McCulloch M., Falter J., Trotter J., Montagna P. (2012). Coral resilience to ocean acidification and global warming through pH up-regulation. Nat. Clim. Change.

[5] Marshall A.T., Clode P.L., Russell R., Prince K., Stern R. (2007). Electron and ion microprobe analysis of calcium distribution and transport in coral tissues. J. Exp. Biol..

[6] Inoue M., Gussone N., Koga Y., Iwase A., Suzuki A., Sakai K. (2015). Controlling factors of Ca isotope fractionation in scleractinian corals evaluated by temperature, pH and light controlled culture experiments. Geochim. Et. Cosmochim. Acta.

[7] Clode P.L., Alan arshall T.M. (2004). Calcium localisation by X-ray microanalysis and fluorescence microscopy in larvae of zooxanthellate and azooxanthellate corals. Tissue Cell.

[8] Venn A., Tambutté E., Holcomb M., Segonds N., Techer N., Zoccola D., D (2011). Live tissue imaging shows reef corals elevate pH under their calcifying tissue relative to seawater. PLoS ONE.

[9] Venn A.A., Tambutté E., Holcomb M., Laurent J., Allemand D., Tambutté S. (2013). Impact of seawater acidification on pH at the tissue-skeleton interface and calcification in reef corals. Proc. Natl. Acad. Sci. USA.

[10] Tambutté E., Venn A.A., Holcomb M., Segonds N., Techer N., Zoccola D., Allemand D. (2015). Morphological plasticity of the coral skeleton under CO_2_-driven seawater acidification. Nat. Commun..

[11] Wilson C.A., Beckman D.W., Dean J.M. (1987). Calcein as a fluorescent marker of otoliths of larval and juvenile fish, Trans. Am. Fish. Soc..

[12] Bernhard J.M., Blanks J.K., Hintz C.J., Chandler G.T. (2004). Use of the fluorescent calcite marker calcein to label foraminiferal tests. J. Foraminifer. Res..

[13] Tambutté E., Tambutté S., Segonds N., Zoccola D., Venn A., Erez J. (2012). Calcein labelling and electrophysiology: insights on coral tissue permeability and calcification. Proc. Biol. Sci..

[14] Shapiro O.H., Kramarsky-Winter E., Gavish A.R., Stocker R., Vardi A. (2016). A coral-on-a-chip microfluidic platform enabling live-imaging microscopy of reef-building corals. Nat. Commun..

[15] Holcomb M., Cohen A.L., McCorkle D.C. (2013). An evaluation of staining techniques for marking daily growth in scleractinian corals. J. Exp. Mar. Biol. Ecol..

[16] Herrmann M., Lepore M.L., Laudien J., Arntz W.E., Penchaszadeh P.E. (2009). Growth estimations of the Argentinean wedge clam *Donax hanleyanus*: a comparison between length-frequency distribution and size-increment analysis. J. Exp. Mar. Biol. Ecol..

[17] Riascos J., Guzman N., Laudien J., Heilmayer O., Oliva M. (2007). Suitability of three stains to mark shells of *Concholepas concholepas* (Gastropoda) and *mesodesma donacium* (Bivalvia). J. Shellfish Res..

[18] Dissard D., Nehrke G., Reichart G.J., Nouet J., Bijma J. (2009). Effect of the fluorescent indicator calcein on Mg and Sr incorporation into foraminiferal calcite. Geochem. Geophys. Geosyst..

[19] Kurtarkar S.R., Saraswat R., Nigam R., Banerjee B., Mallick R., Naik D.K., Singh D.P. (2015). Assessing the effect of calcein incorporation on physiological processes of benthic foraminifera. Mar. Micropaleontol..

[20] Inoue M., Shinmen K., Kawahata H., Nakamura T., Tanaka Y., Kato A. (2012). Estimate of calcification responses to thermal and freshening stresses based on culture experiments with symbiotic and aposymbiotic primary polyps of a coral, *Acropora digitifera*. Glob. Planet. Change.

[21] Moya A., Huisman L., Ball E.E., Hayward D.C., Grasso L.C., Chua C.M. (2012). Whole transcriptome analysis of the coral *Acropora millepora* reveals complex responses to CO₂-driven acidification during the initiation of calcification. Mol. Ecol..

[22] Nakajima Y., Nishikawa A., Iguchi A., Sakai K. (2010). Gene flow and genetic diversity of a broadcast-spawning coral in northern peripheral populations. PLoS ONE.

[23] Hayashibara T., Shimoike K. (2002). Cryptic species of *Acropora digitifera*. Coral Reefs.

[24] Morita M., Nishikawa A., Nakajima A., Iguchi A., Sakai K., Takemura A. (2006). Eggs regulate sperm flagellar motility initiation, chemotaxis and inhibition in the coral *Acropora digitifera*, *A. gemmifera* and *A. tenuis*. J. Exp. Biol..

[25] Iwao K., Fujisawa T., Hatta M. (2002). A cnidarian neuropeptide of the GLWamide family induces metamorphosis of reef-building corals in the genus *Acropora*. Coral Reefs.

[26] Hirose M., Yamamoto H., Nonaka M. (2007). Metamorphosis and acquisition of symbiotic algae in planula larvae and primary polyps of *Acropora* spp. Coral Reefs.

[27] Ainsworth T.D., Fine M., Blackall L.L., Hoegh-Guldberg O. (2006). Fluorescence *in situ* hybridization and spectral imaging of coral-associated bacterial communities. Appl. Environ. Microbiol..

[28] Gilis M., Meibom A., Alexander D., Grauby O., Stolarski J., Baronnet A. (2015). Morphology, microstructure, crystallography, and chemistry of distinct CaCO_3_ deposits formed by early recruits of the scleractinian coral *Pocillopora damicornis*. J. Morphol..

[29] Gilis M., Meibom A., Domart-Coulon I., Grauby O., Stolarski J., Baronnet A. (2014). Biomineralization in newly settled recruits of the scleractinian coral *Pocillopora damicornis*. J. Morphol..

[30] Nielsen M.H., Shaul A., De Yoreo J.J. (2014). In situ TEM imaging of CaCO_3_ nucleation reveals coexistence of direct and indirect pathways. Science.

