# Supplementary material for: Calcification process dynamics in coral primary polyps as observed using a calcein incubation method
Source: Biochem Biophys Rep. 2017 Jan 24;9:289–94. doi: 10.1016/j.bbrep.2017.01.006 (PMC5627507; doi:10.1016/j.bbrep.2017.01.006)
Supplement: Supplementary material [file mmc5.docx]

There are no conflicts of interest to declare.
