# Supplementary material for: Calcification process dynamics in coral primary polyps as observed using a calcein incubation method
Source: Biochem Biophys Rep. 2017 Jan 24;9:289–94. doi: 10.1016/j.bbrep.2017.01.006 (PMC5627507; doi:10.1016/j.bbrep.2017.01.006)

**Supplementary Materials and Methods**

***Effects of long-term calcein incubation on coral polyp***

A total of 51 aposymbiotic primary polyps of *Acropora* sp.1 were used to evaluate the effects of calcein on coral primary polyps (Fig. S1 and Fig. S2). The specimens were incubated with FSW-calcein at four different concentrations (0 µM, n = 14; 10 µM, n = 9; 100 µM, n = 15; 500 µM, n = 14) in an incubator (VEROSOS, Hiroshima, Japan) in the dark at 27.0 ± 0.1°C. FSW-calcein was changed daily (between 5 and 10 pm) with new solutions to maintain stable pH and oxygen content. After 10 days, soft primary polyp tissues were removed using a water-pick. All individual primary poly skeletons were dried and subsequently weighed with a microbalance (Cahn C-34, California, USA) (Inoue et al. 2012 [1]) (Fig. S1). Whole picture of primary polyps were acquired using a Keyence VHX-1000 digital microscope (Osaka, Japan) (Fig. S2). Areas of primary polyp occupancy were measured using ImageJ software (US National Institutes of Health, Bethesda, MD, USA). Skeletal microstructure images were obtained by a scanning electron microscope (S-3000N; HITACHI, Tokyo, Japan) after sputtering thin platinum film in the sputtering apparatus. We evaluated the effects of long-term incubation with calcein on aposymbiotic primary polyps. Only one of 14 primary polyps grown under control conditions had died, whereas all polyps grown under calcein-FSW conditions were alive, indicating that calcein had a negligible effect on the survival of coral primary polyps.

Conversely, the inhibition of skeletal formation, indicated by a lack of branch tips (circles in Fig. S1A), was observed via bright field observation at a calcein concentration of 500 µM (white arrows in Fig. S1D). *Acropora* corals form regular shingles on the skeletal surface and the shingles are aligned along the extensional direction of the structures and cover the entire skeletal surface [2]. By adding calcein-FSW solutions, these shingles were gradually lost and the skeletal surface exhibited clear changes in a dose-dependent manner (Fig. S1E–H). Shingles on the skeletal surface of untreated samples were regularly aligned whilst those treated with 500-µM calcein abnormal smooth surfaces. No evident effects of the calcein concentration on areas of primary polyp occupancy were observed (Fig. S2A; one-way ANOVA: *F*_1,45_ = 0.61, *p* >0.1). In contrast, the skeletal weights differed significantly among treatments (Fig. S2B; one-way ANOVA: *F*_1,45_ = 8.50, *p* <0.01). In particular, 500-µM calcein had an evident effect on the coral skeletal weight (Fig. S2B). These results suggested that a high calcein concentration affected skeletal formation and decreased the skeletal weight. However, the effects on areas of primary polyp occupancy were unclear, suggesting that calcein only inhibits vertical growth of the primary polyp skeleton. Because a lower concentration of calcein (<100 µM) did not affect skeletal formation in primary polyps, we set the calcein concentration at 100 µM for the following analyses.

**Supplementary Figure Legends**

**Supplementary Fig. 1.** Microstructures in an aposymbiotic primary polyp with calcein incubation. **(A–D)** Bright-field image of aposymbiotic primary polyp skeletons exposed to different concentrations of calcein. Scale bar: 200 µm. **(E–F)** Scanning electron microscopy images of branch tips (e.g., yellow circles in **(A)**). Yellow allow indicate a shingle of the skeletal surface. Scale bar: 20 µm. **(A, E** Non-treated primary polyp; **(B, F)** Treated using 10 µM, **(C, G)** 100 µM, or **(D, H)** 500 µM calcein. Circles indicate inhibited skeletal formation (e.g., lack of branch tips). White arrow indicates inhibited extension at branch tips.

**Supplementary Fig. 2.** Mean areas of occupation **(A)** and mean skeletal weights **(B)** of primary polyps of *Acropora* sp.1 under four calcein concentrations. Bars indicate means and standard deviations (n = 13 for 0 µM; n = 9 for 10 µM; n = 15 for 100 µM; n = 10 for 500 µM).

**Supplementary Fig. 3. (A)** Confocal observation of coral crystals. Red arrows show the positions of calcification centers. Scale bar: 10 µm. **(B)** Magnified images of the dotted square area in **(A)**. White arrows show the positions of cracks in the crystals. Values in each panel indicate the vertical position from the bottom. Scale bar: 5 µm.

**Supplementary Fig. 4. (A)** Calcein staining pattern in tissues during time-lapse imaging. In the beginning of the experiment, no green fluorescence was observed. Calcein-FSW was added 10 sec after starting the experiment, and after 130 sec, some of the crystals were stained with calcein (white arrow). Yellow arrows indicate the subcalicoblastic medium. Scale bar: 100 µm. **(B)** Images of dumbbell- and rod-shaped crystals 24 h after Hym-248 addition. Calcein was added 1 h prior to the observations. At that time, no green fluorescence was observed from the coral tissue. Dumbbell-shaped crystals are located on the left sides of the images. The numbers in the upper images indicate the sampling times. Scale bar: 10 µm. **(C)** High-magnification image of the area enclosed by a white dotted line in **(B)**. White arrow indicates a putative smaller crystal. Yellow arrow indicates a calicoblastic cell (black). Scale bar: 3 µm. All of the images were captured at the surface of the glass-based dish

**Refarences**

1. M. Inoue, K. Shinmen, H. Kawahata, T. Nakamura, Y. Tanaka, A. Kato, *et al*. Estimate of calcification responses to thermal and freshening stresses based on culture experiments with symbiotic and aposymbiotic primary polyps of a coral, *Acropora digitifera*, Glob. Planet. Change. 92 (2012) 1–7.
2. J. Stolarski, F.R. Bosellini, C.C. Wallace, A.M. Gothmann, M. Mazur, I, Domart-Coulon, *et al*. A unique coral biomineralization pattern has resisted 40 million years of major ocean chemistry change. Sci Rep. (2016) 6.


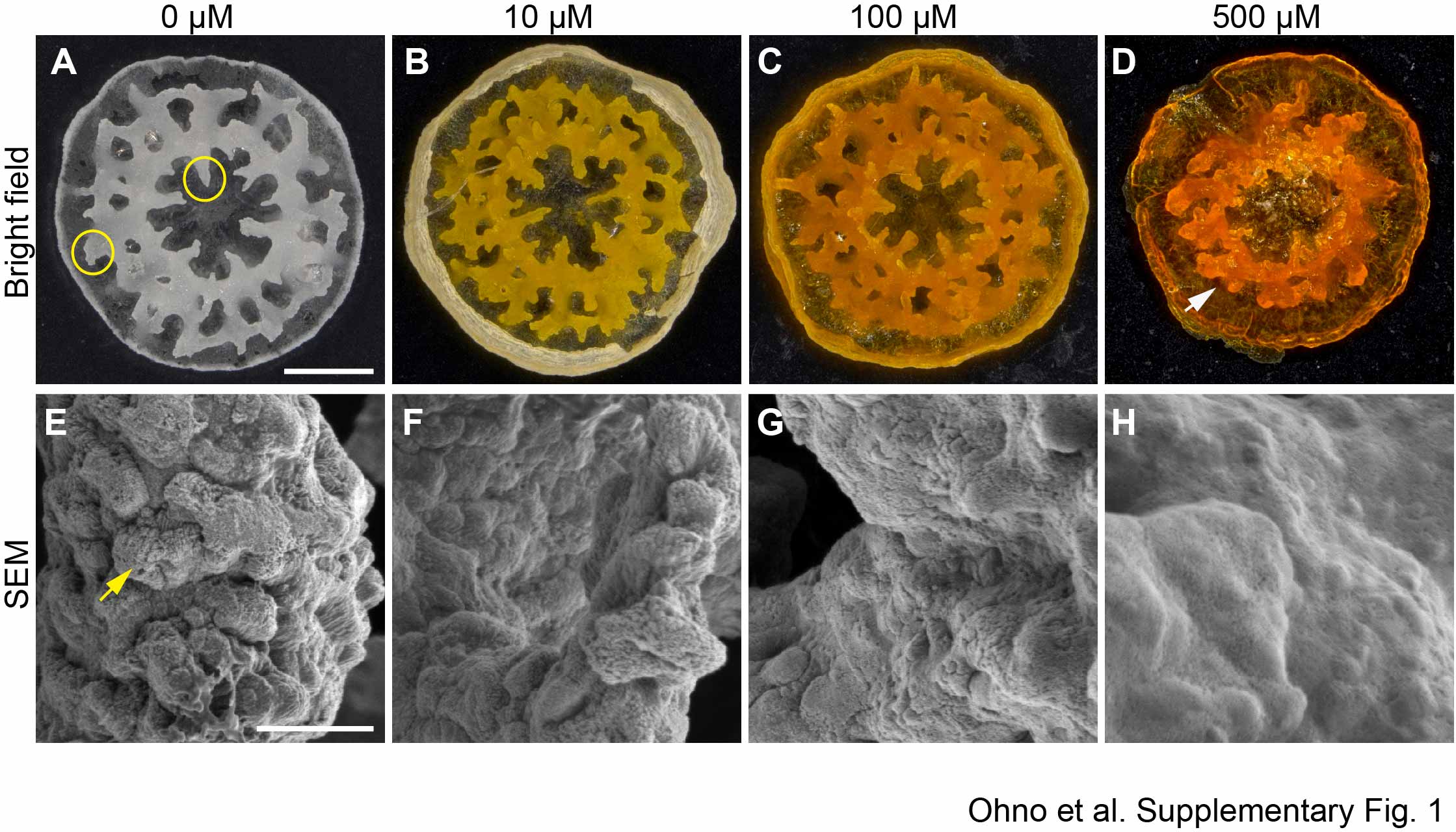


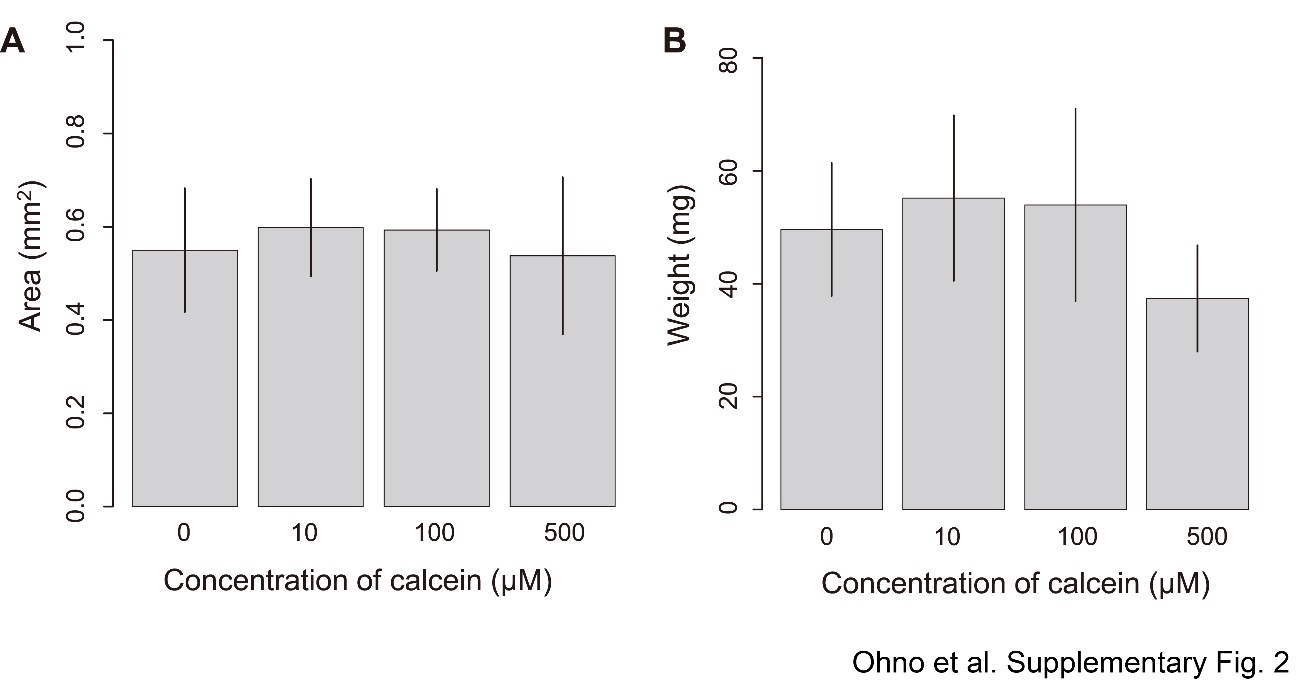


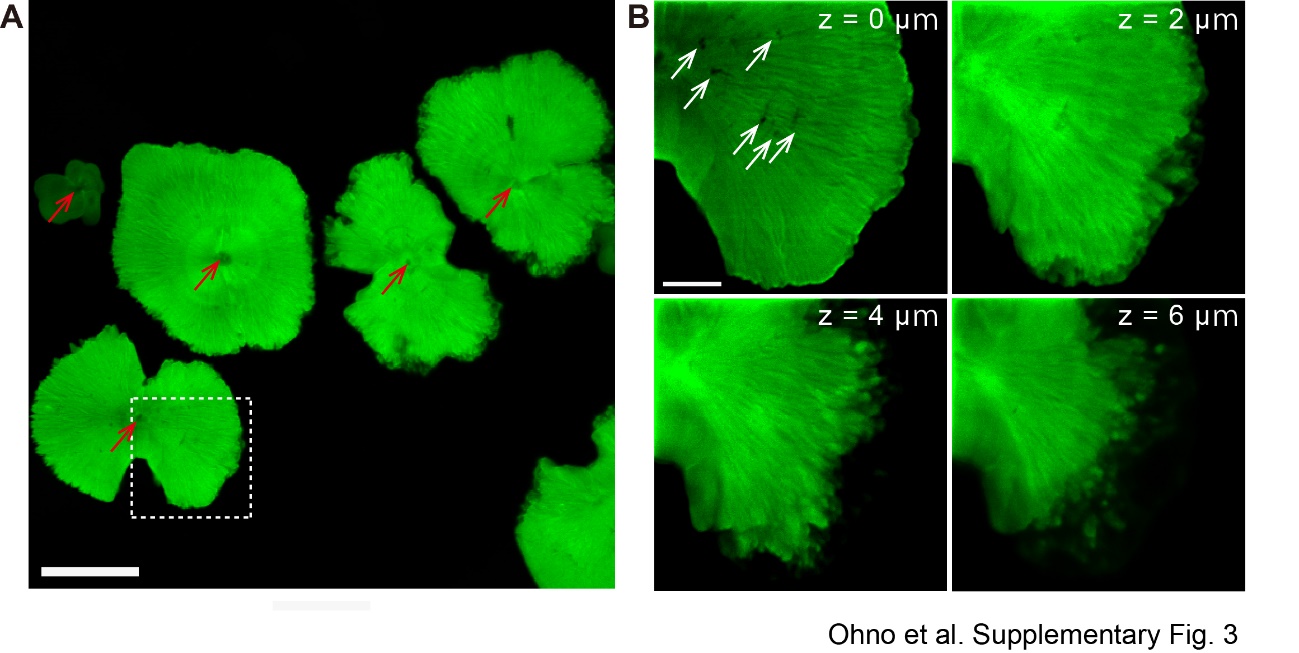

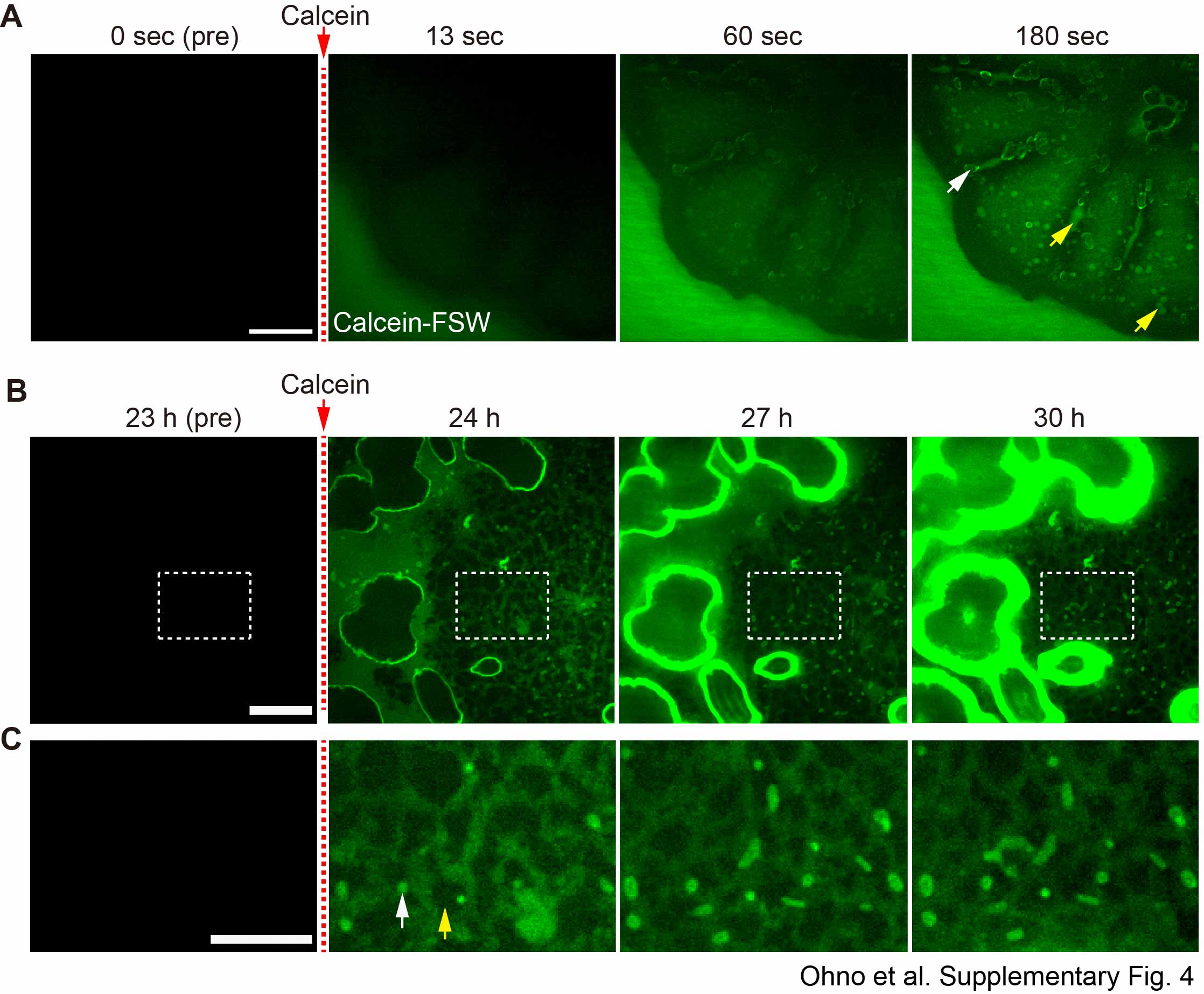

Supplement: Supplementary material [file mmc4.docx]
